# Nicotine regulates activity of lateral habenula neurons via presynaptic and postsynaptic mechanisms

**DOI:** 10.1038/srep32937

**Published:** 2016-09-06

**Authors:** Wanhong Zuo, Cheng Xiao, Ming Gao, F. Woodward Hopf, Krešimir Krnjević, J. Michael McIntosh, Rao Fu, Jie Wu, Alex Bekker, Jiang-Hong Ye

**Affiliations:** 1 Department of Anesthesiology, Pharmacology and Physiology, Rutgers, The State University of New Jersey, New Jersey Medical School, Newark, New Jersey, USA; 2 Divisions of Neurology, Barrow Neurological Institute, St. Joseph’s Hospital and Medical Center, Phoenix, AZ, USA; 3 Department of Neurology, University of California at San Francisco, CA, USA; 4 Department of Physiology, McGill University, Montréal, Canada; 5 George E. Wahlen Veterans Affairs Medical Center and Departments of Psychiatry and Biology, University of Utah, Salt Lake City, UT, USA

## Abstract

There is much interest in brain regions that drive nicotine intake in smokers. Interestingly, both the rewarding and aversive effects of nicotine are probably critical for sustaining nicotine addiction. The medial and lateral habenular (LHb) nuclei play important roles in processing aversion, and recent work has focused on the critical involvement of the LHb in encoding and responding to aversive stimuli. Several neurotransmitter systems are implicated in nicotine’s actions, but very little is known about how nicotinic acetylcholine receptors (nAChRs) regulate LHb activity. Here we report in brain slices that activation of nAChRs depolarizes LHb cells and robustly increases firing, and also potentiates glutamate release in LHb. These effects were blocked by selective antagonists of α6-containing (α6*) nAChRs, and were absent in α6*-nAChR knockout mice. In addition, nicotine activates GABAergic inputs to LHb via α4β2-nAChRs, at lower concentrations but with more rapid desensitization relative to α6*-nAChRs. These results demonstrate the existence of diverse functional nAChR subtypes at presynaptic and postsynaptic sites in LHb, through which nicotine could facilitate or inhibit LHb neuronal activity and thus contribute to nicotine aversion or reward.

Nicotine, a major bioactive component in tobacco that drives addiction to smoking, has both strong rewarding and aversive effects^1^. Although reward has been considered important for addiction, both effects may be critical for promoting smoking, since nicotine aversion can promote craving for nicotine and intake in addicted individuals^1^,^2^. It is well-known that nicotine reinforcement involves midbrain dopaminergic relays to nucleus accumbens^3^, but the circuitry mediating aversive effects is less clear. Several lines of evidence suggest that nicotine may induce reward and aversion through opposite actions on the mesolimbic system^4^,^5^. In addition, recent studies of aversion have focused on the lateral and medial habenula (LHb and MHb)^1^. The MHb is known to negatively regulate nicotine intake and contribute to nicotine aversion^1^,^6^,^7^,^8^. There is also much interest in the LHb; LHb neurons are activated by several aversive stimuli^9^,^10^, which in turn excites GABAergic neurons in the rostromedial tegmental nucleus (RMTg)^9^ that inhibit midbrain dopaminergic neurons^11^,^12^. In this way, LHb could encode aversion to unpleasant stimuli^13^,^14^ as well as nicotine aversion^1^,^15^. Although the LHb is critical for both nicotine reinstatement^16^ and nicotine related anxiety^15^, little is known about the mechanisms through which nicotine regulates LHb neuronal activity. Previous work has shown that nicotine robustly excites RMTg neurons^12^, perhaps indirectly by stimulating the LHb.

Nicotine acts primarily through nicotinic acetylcholine receptors (nAChRs) composed of multiple subunits, which are present throughout the brain including in LHb^17^ and MHb^18^. Studies from transgenic mice suggest that many nAChR subtypes can contribute to nicotine withdrawal, reinforcement and aversion^1^,^19^,^20^. α6*-containing nAChRs in particular are highly expressed in mesolimbic pathways and mediate nicotine-related reward^21^,^22^,^23^, though the relevant brain locations have not been completely identified^21^,^24^,^25^. Given the prominent aversive effects of nicotine^1^ and the LHb’s importance for aversion, we used a combination of electrophysiology and pharmacology approach and α6*-knockout mice to investigate whether different nAChRs contribute to nicotine modulation of LHb neuronal activity.

## Results

### Nicotine excites LHb neurons

**Figure 1:**
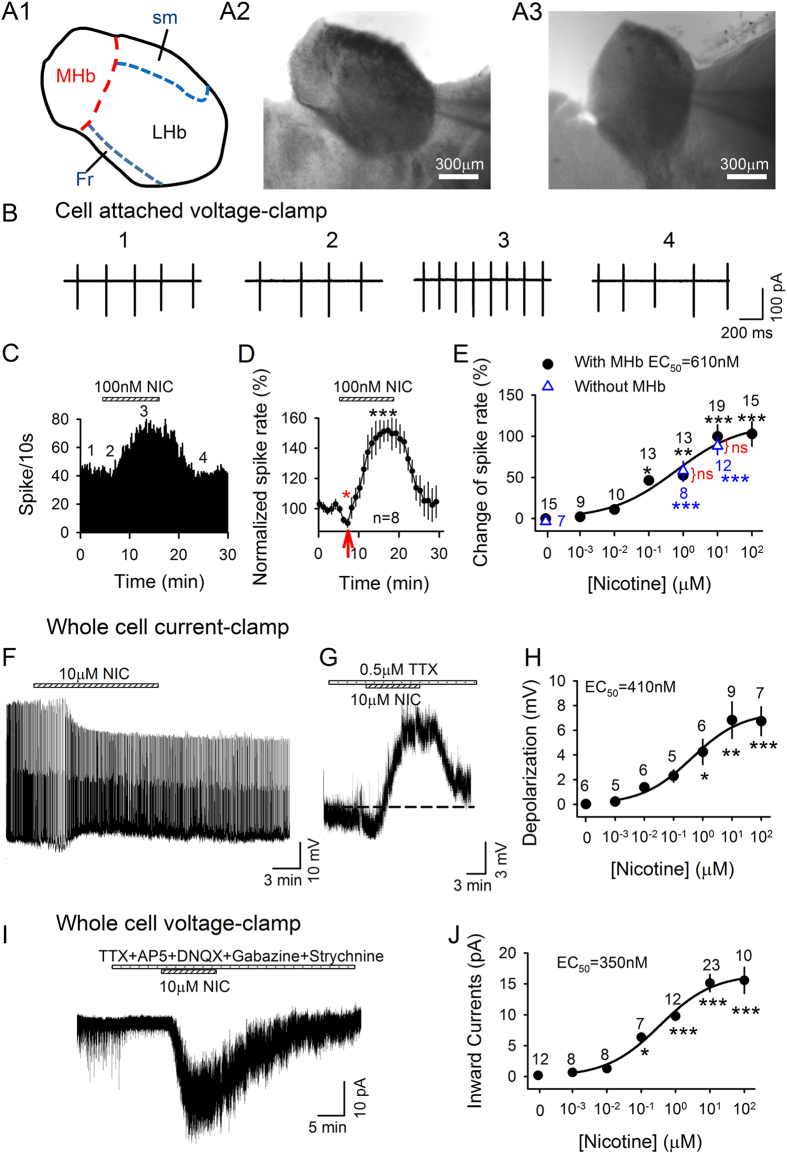
Nicotine regulates LHb activity. (**A**) Brain atlas diagram for approximate location of MHb and LHb (**A1**). Representative images of coronal slices containing the LHb with (**A2**) and without MHb (**A3**). (**B–D**) Nicotine first slowed and then accelerated LHb firing. Red arrow: initial firing reduction; (**E**) Concentration-response of nicotine-induced % increase of firing rate, recorded in cell-attached mode, in brain slices containing both LHb and MHb (●). Nicotine (1 μM and 10 μM) caused similar increases in firing rate of LHb neurons in brain slices with removed MHb (∆). ns = no significance, with MHb vs without MHb. Number of neurons is indicated. Nicotine depolarization of LHb neurons (**F**), even in presence of TTX (**G,H**). (**I,J**) Nicotine-induced inward currents at a holding potential of −70 mV, and in the presence of Inhibitor Cocktail. *p < 0.05, **p < 0.01, ***p < 0.001, one-way ANOVA.

As reported previously^26^,^27^, there is a clear border between the LHb and the adjacent MHb (Figs 1A1 and 2). We thus distinguished the MHb and LHb neurons according to the clear border. In addition, consistent with previous reports, we observed that LHb neurons were loosely dispersed^26^, had heterogeneous morphological but similar membrane properties^27^, and were capable of producing burst firing^26^,^28^.

In the current study, we recorded from ~750 LHb neurons. Nicotine increased firing in both cell-attached (Fig. 1B–D) and whole-cell modes (Fig. 1F). Nicotine accelerated spontaneous firing of 77/95 LHb neurons in a dose-dependent manner, with an EC_50_ of 610 nM (*F*_6,87_ = 15.2, *p* < 0.001), and reached the plateau at 10 μM (Fig. 1B–E). Interestingly, upon bath application of 100 nM nicotine, firing first decreased (to 90.4 ± 3.8% of baseline, *p* < 0.05; Fig. 1D), then sharply accelerated (*p* < 0.001), returning to baseline level after washout (Fig. 1B–D). To confirm that the site of nicotine action was within the LHb, recordings in a subset of experiments were made from slices with MHb removed (Fig. 1A3). Nicotine acceleration of LHb neuron firings in these slices (*F*_2,24_=19.9, *p* < 0.001; *post hoc p* < 0.001, Fig. 1E blue triangle) was similar to that with MHb (*p* > 0.5 with MHb vs without MHb).

In agreement with nicotine enhancement of firing, in the presence of tetrodotoxin (TTX), nicotine also dose-dependently depolarized LHb neurons under current-clamp mode (Fig. 1F–H), with an EC_50_ of 410 nM (*F*_6,37_ = 8.2, *p* < 0.001; Fig. 1H). Similarly, nicotine induced inward currents (Fig. 1I) in 68/81 neurons under voltage-clamp at −70 mV (in presence of blockers of sodium channels, GABA_A_, glycine, NMDA- and AMPA-type-glutamate receptors) which were dose-dependent (*F*_6,73_ = 27.5, *p* < 0.001; Fig. 1J) with an EC_50_ of 350 nM. Thus, nicotine depolarized LHb neurons and increased their firing.

**Figure 2:**
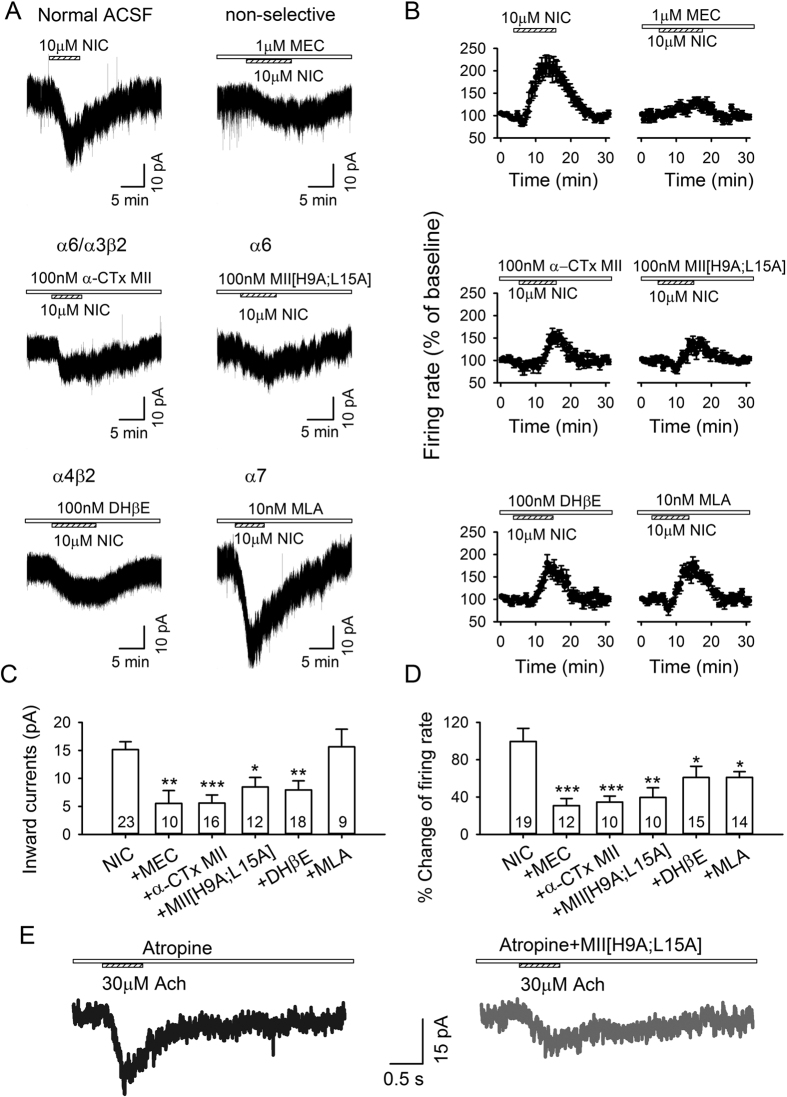
nAChR subtypes mediating nicotine effects in LHb. (**A**) Nicotine-induced inward currents were blocked by MEC, α-CTx-MII, MII[H9A; L15A] and DHβE, but not MLA. (**B**) Nicotine-induced increases in firing rate was prominently reduced by MEC, and MII [H9A; L15A], and weakly but significantly reduced by MLA. (**C,D**) Summary of above data. (**E**) Local ACh application induced an MII [H9A; L15A]-sensitive inward current. *p < 0.05, **p < 0.01, ***p < 0.001.

To identify nAChR subtypes that mediated nicotinic excitation of LHb neurons, we examined the effects of several nicotine antagonists on nicotine-induced inward currents and on firing. Since 10 μM nicotine produced the maximal effect on LHb neuronal activity, we compared the peak amplitude of inward currents induced by 10 μM nicotine in the absence and presence of various nAChR antagonists, which were applied for 5–8 min before co-applying nicotine or ACh. Nicotine currents were greatly reduced (*F*_5,82_ = 6.5, *p* < 0.001, One-way ANOVA; Fig. 2A,C) by mecamylamine (MEC, 1 μΜ, *p* = 0.002), a non-selective nAChR antagonist, α-CTx-MII (100 nM, *p* < 0.001), an selective α6* and α3β2-nAChRs antagonist^22^,^29^, MII[H9A; L15A] (100 nM, *p* = 0.036), an α6*-nAChR blocker^30^, dihydro-beta-erythroidine (DHβE, 100 nM, *p* = 0.006), relatively selective for α4β2-nAChRs, but not methyllycaconitine (MLA, 10 nM), an α7-nAChR antagonist (*p* > 0.5). Thus, α-CTx-MII-sensitive (α6*- ± α3β2-) and DHβE-sensitive (α4β2) nAChRs contributed to the nicotine-induced LHb depolarization (Fig. 2C). In addition, although 10 μM nicotine is higher than occurs physiologically in human smokers^31^, this concentration of nicotine was maximal and thus useful for determining in impact of different nAChR antagonists on nicotine modulation of different aspects of LHb physiology.

We then examined the nAChR subtypes which mediated the nicotine enhancement of LHb firing. Nicotine enhancement of firing was greatly attenuated by the nAChR antagonists tested above (*F*_5,70_ = 6.2, *p* < 0.001; Fig. 2B,D). *Post hoc* tests revealed substantial inhibition by MEC (*p* < 0.001), α-CTx-MII (*p* < 0.001) and MII [H9A; L15A] (*p* = 0.001), while DHβE (*p* = 0.034) and MLA (*p* = 0.049) produced a smaller inhibition. Thus, MII-sensitive (α6*- ± α3β2)–nAChRs played a prominent role in both the nicotine-induced firing enhancement (Fig. 2D) and membrane depolarization, while DHβE-sensitive (α4β2) and MLA-sensitive (α7) subtypes had partial and dissociable effects.

Apart from direct effects on LHb neurons, bath-applied nicotine could act indirectly by stimulating cholinergic MHb neurons (~40% of which express α6*-nAChR mRNA^32^) that project to the LHb. To minimize indirect effects through MHb, we locally applied ACh (30 μΜ) onto the somata of recorded LHb neurons by pressure injection from a second micropipette^33^. In the presence of atropine, ACh induced an inward current (25.6 ± 7.1 pA) in 6/10 neurons tested (*p* = 0.005) which was significantly depressed by MII [H9A; L15A] (to 33.0 ± 4.0% of ACh, *p* = 0.016; Fig. 2E). These experiments confirmed that there was a direct, MII [H9A; L15A]-sensitive nAChR (likely α6*-nAChR)-mediated depolarization of LHb cells. Indirect effects via MHb were also unlikely since nicotine depolarized LHb cells (Figs 1F–H and 2A–D) in the presence of TTX and glutamate and GABA antagonists, which would block the corresponding synaptic inputs^11^,^34^,^35^ from MHb neurons, and also because nicotine activated LHb neurons in brain slices where MHb was cut off (Fig. 1E).

### Nicotine enhances synaptic potentials and currents (IPSCs and EPSCs) in LHb neurons

**Figure 3:**
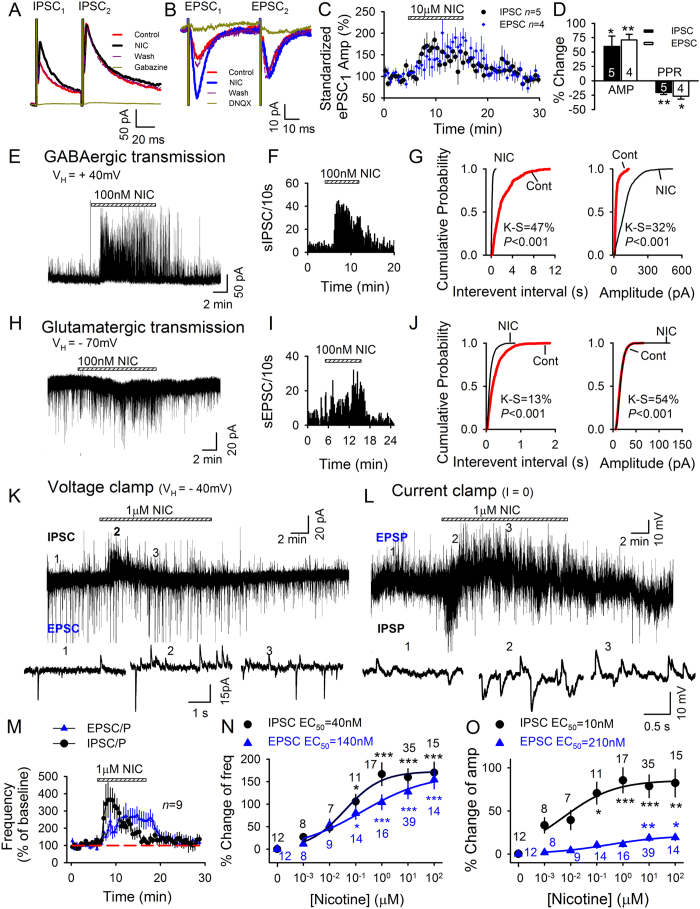
Nicotine enhances IPSCs and EPSCs in LHb neurons. (**A,B**) Nicotine action on paired evoked IPSCs (eIPSCs) (**A**) and evoked EPSCs (eEPSCs) (**B**). eIPSCs (recorded at V_H_ = +40 mV) were abolished by gabazine (10 μM), a GABA_A_ receptor antagonist; eEPSCs (at V_H_ = −70 mV) were abolished by DNQX (20 μM), an AMPA receptor blocker. (**C**) Nicotine (10 μM) enhanced the first of PSC pairs. (**D**) % change in amplitude (AMP) of first PSCs and paired pulse ratio (PPR = PSC_2_/PSC_1_). (**E–J**) Nicotine increased spontaneous sIPSC frequency and amplitude (**E–G**) as well as sEPSC frequency (**H–J**). (**K,L**) Simultaneous recording of sIPSC/sIPSPs and sEPSC/sEPSPs at −40 mV under voltage-(**K**) and current-clamp (**L**). (**M**) Different time course of nicotine action on the frequency of sIPSC/Ps and sEPSC/Ps (*n* = 9). (**N,O**) Concentration-dependence of increased sEPSC and sIPSC frequency (**N**) and amplitude (**O**). *p < 0.05, **p < 0.01, ***p < 0.001.

We next examined whether nicotine would alter inhibitory and excitatory post-synaptic currents (IPSCs and EPSCs, respectively) in LHb neurons. We first examined how nicotine affects paired-pulse transmission when two PSCs were evoked at a brief inter-stimulus interval (50 msec). Nicotine (10 μΜ) selectively enhanced the first IPSC of each pair (IPSC_1_; *p* < 0.05; Fig. 3A,C), thus reducing the paired-pulse ratio (PSC_2_/PSC_1_ = PPR) (*p* < 0.01; Fig. 3D). Similarly, nicotine enhanced only EPSC_1 _of each pair (*p* < 0.01; Fig. 3B–D) and reduced the EPSC-PPR (*p* < 0.05). Since decreased PPR can reflect increased transmitter release at the synapse, these results suggest that nicotine might potentiate synaptic transmission in LHb by increasing presynaptic release of both glutamate and GABA.

To better understand the impact of nicotine on GABA and glutamate signaling in the LHb, we then tested the effects of nicotine on spontaneous synaptic currents. Nicotine induced a transient shift to higher frequencies and larger amplitudes of spontaneous IPSCs (sIPSCs) (Fig. 3E–G), with an EC_50_ of 40 nM for frequency (*F*_6,98_ = 9.2, *p* < 0.001; Fig. 3N, black line) and 10 nM for amplitude (*F*_6,98_ = 3.9, *p* = 0.002; Fig. 3O, black line). By contrast, nicotine induced a slowly developing increase in spontaneous EPSC (sEPSC) frequency (Fig. 3H,I) with an EC_50_ of 140 nM (*F*_6,105_ = 8.9, *p* < 0.001; Fig. 3N, blue line), and a small but significant increase in sEPSC amplitude (*p* < 0.001, Kolmogorov-Smirnov test; Fig. 3J) with an EC_50_ of 210 nM (*F*_6,105_ = 4.9, *p* < 0.001; Fig. 3O, blue line). At a holding potential of −40 mV, sEPSCs and sIPSCs appeared as distinct inward and outward currents, and nicotine (1 μΜ) induced a transient outward current followed by a sustained inward current and increased frequency of sIPSCs and sEPSCs (Fig. 3K,M). Similar changes were seen under current clamp, with an initial hyperpolarization followed by depolarization and increased frequency of sIPSPs and sEPSPs (Fig. 3L,M). Thus, nicotine has a biphasic action in LHb, causing a transient enhancement of ongoing GABA release and a slower but sustained increase in glutamate release.

**Figure 4:**
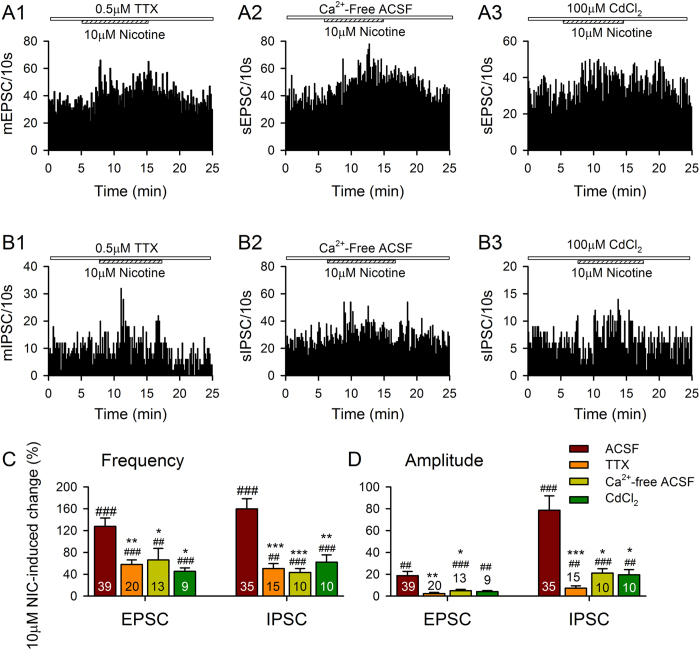
Nicotine causes a smaller but significant increase in the frequency of spontaneous EPSCs (**A1–3**) and IPSCs (**B1–3**) in 0.5 μM TTX, in calcium free ACSF (extracellular Ca^2+^ was replaced with Mg^2+^), and in 100 μM CdCl_2_ (calcium channel blocker). (**C,D**) % increases in sEPSC and sIPSC frequency (**C**) and amplitude (**D**) induced by nicotine in the absence (ACSF) and presence of TTX, or calcium free ACSF, or CdCl_2_. ^##^*p* < 0.01, ^###^*p* < 0.001, Student’s paired *t*-test for nicotine application vs pre-nicotine control. *p < 0.05, **p < 0.01, ***p < 0.001, One-way ANOVA followed by Tukey’s multiple comparison test for nicotine application vs nicotine plus TTX/calcium free ACSF/CdCl_2_.

We also examined the impact of nicotine on miniature synaptic currents, where spontaneous release was determined in the presence of TTX, Ca^2+^-free ACSF (where CaCl_2_ was replaced by MgCl_2_) or in presence of 100 μM cadmium chloride (CdCl_2_, a non-selective blocker of voltage-gated calcium channels). All of these treatments allow determination of the effects of nicotine on synaptic release in the absence of possible effects on presynaptic action potential generation (TTX) or activity-dependent effects on release (calcium-free or cadmium-containing media). Nicotine caused a smaller but still significant increase in the frequency of spontaneous miniature EPSCs (*F*_3,77_ = 5.8, *p* = 0.001; *post hoc p* = 0.004) and IPSCs (*F*_3,66_ = 10.2, *p* < 0.001; *post hoc p* < 0.001) in presence of TTX (Fig. 4A1,B1,C), in Ca^2+^-free ACSF (EPSC: *p* =0.042; IPSC: *p* < 0.001; Fig. 4A2,B2,C) or in presence of CdCl_2,_ EPSC: *p* = 0.014; IPSC: *p* = 0.005; Fig. 4A3,B3,C), when compared with the effects of nicotine on spontaneous release when presynaptic activity is allowed to occur. In addition, both miniature EPSC (*F*_3,77_ = 5.3, *p* = 0.002; Fig. 4D) or IPSC (*F*_3,66_ = 7.6, *p* < 0.001; Fig. 4D) amplitudes were significantly reduced in the presence of TTX (EPSC: *p* = 0.003; IPSC: *p* < 0.001) or Ca^2+^-free ACSF (EPSC: *p* = 0.046; IPSC: *p* = 0.018). These results are consistent with both presynaptic and postsynaptic actions of nicotine at excitatory and inhibitory synapses, increasing glutamatergic and GABAergic release, and that these actions depended in part on calcium channels.

**Figure 5:**
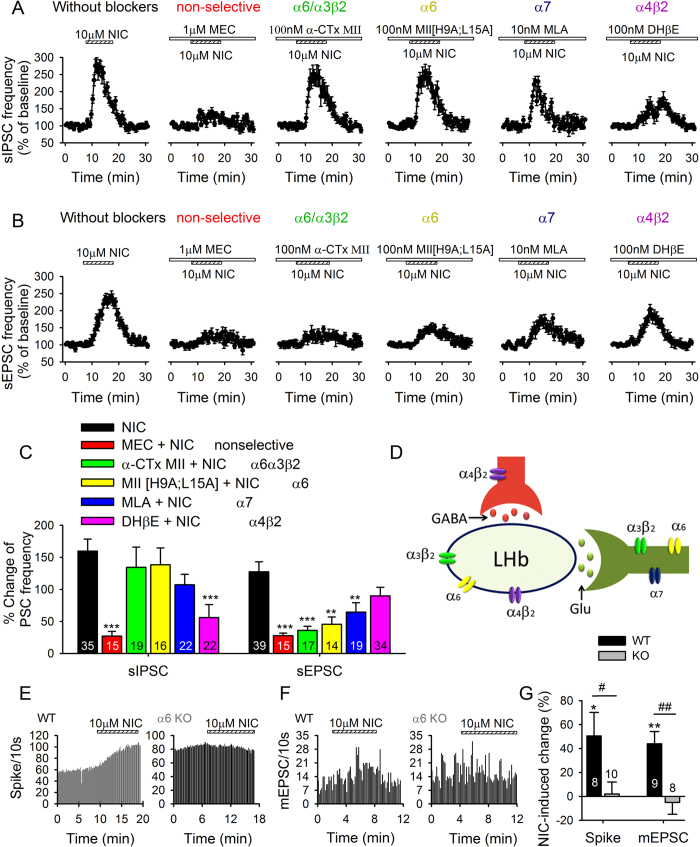
nAChR subtypes mediating nicotine effects on sIPSCs and sEPSCs. (**A,B**) Effects of various nAChR blockers on nicotine-induced acceleration of sIPSCs (**A**) and sEPSCs (**B**). (**C**) Summary of above data. *p < 0.05, **p < 0.01, ***p < 0.001. (**D**) Proposed schematic of nAChR subtype distribution in the LHb. (**E,F**) Nicotine enhanced firing (**E**) and mEPSC frequency (**F**) recorded in brain slices from wild-type (WT) but not α6-nAChR knock out (α6 KO) mice. *p < 0.05, ^#^*p* < 0.05, ^##^*p* < 0.01. (**G)** Summary of above data.

We next examined the nAChR subtypes that mediate the nicotinic enhancement of spontaneous synaptic currents. Increased sIPSC frequency was greatly attenuated (*F*_5,123_ = 5.5, *p* < 0.001; Fig. 5A,C) by MEC (*p* < 0.001) and DHβE (*p* < 0.001) but not α-CTx-MII (*p* > 0.5), MII[H9A; L15A] (*p* > 0.5) or MLA (*p* = 0.28). Antagonists had no effects when applied alone (data not shown). Thus, DHβE-sensitive (α4β2)-nAChRs but not α6*-nAChRs were important for mediating the nicotinic enhancement of sIPSC frequency. In contrast, the enhancement of sEPSC frequency was reduced (*F*_5,132_ = 7.4, *p* < 0.001; Fig. 5B,C) by MEC (*p* < 0.001), α-CTx-MII (*p* < 0.001), MII[H9A; L15A] (*p* = 0.001), less by MLA (*p* = 0.009), but not significantly by DHβE (*p* = 0.125). Thus, MII-sensitive (α6* ± α3β2)- and MLA-sensitive (α7)-nAChRs mediated nicotine’s effects on glutamatergic inputs to LHb neurons, similar to what was observed for the nicotine enhancement of LHb firing (Fig. 2H).

### Increased firing and mEPSC frequency by nicotine are absent in α6-nAChR knock-out

Since pharmacology experiments suggested that α6*-nAChRs were critical for nicotine actions in the LHb (Fig. 2), we directly tested the importance of α6-nAChRs by comparing nicotine actions on LHb neurons from wild-type (WT) and α6-nAChR knock-out (α6-KO) mice. Compared to the WT mice, LHb neurons in the α6-KO mice exhibit higher basal firing rate (WT: 6.6 ± 1.4 Hz, α6-KO: 11.8 ± 2.1 Hz; *p* = 0.043 WT vs KO) and basal mEPSC frequency (WT: 1.0 ± 0.1 Hz; α6-KO: 2.0 ± 0.5 Hz; *p* = 0.045) but not basal mEPSC amplitude (WT: 12.5 ± 0.7 pA; α6-KO: 12.3 ± 0.7 pA; *p* = 0.84). Importantly, nicotine (10 μM) significantly increased LHb firing in WT but not α6-KO mutants (*p* = 0.03 WT vs KO; Fig. 5E,G), and increased LHb mEPSC frequency in WT but not α6-KO mutants (*p* = 0.004; Fig. 5F,G). These results directly demonstrate the importance of α6*-nAChRs for the nicotine-induced enhancement of LHb activity. Alternatively, these results could reflect an occlusion of the ability of nicotine to increase firing in α6-KO mutants which have a higher basal firing rate.

### Mechanism of nicotine’s biphasic action on LHb neurons

As described in Fig. 1, upon the application of 100 nM nicotine, LHb firing was first decreased; and then sharply accelerated, returning to baseline after washout (Fig. 1A–C). We proposed that the decrease of firing was mediated by nicotinic potentiation of GABAergic transmission, and the enhancement of firing was mediated in part by nicotinic potentiation of glutamatergic transmission. To test this hypothesis, we compared the effects of 100 nM nicotine in the absence and presence of a GABA_A_ receptor antagonist (20 μM gabazine), glutamate receptor antagonists (20 μM DNQX plus 50 μM AP5), or a cocktail of both GABA_A_ and glutamate receptor blockers (gabazine + DNQX +AP5).

As shown in Table 1, nicotine-induced inhibition of firing was completely abolished (*F*_3,28_ = 7.7, *p* < 0.001) by gabazine (*p* = 0.042) or by the cocktail (*p* = 0.011), but not by DNQX +AP5 (*p* > 0.5). In contrast, nicotine-induced enhancement of firing was partly attenuated (*F*_3,34_ = 5.9, *p* = 0.002) by DNQX +AP5 (*p* = 0.013) or by the cocktail (*p* = 0.04), but not by gabazine alone (*p* > 0.5). Thus, the ability of nicotine to increase and decrease firing was mediated by nicotine enhancement of IPSCs and EPSCs, respectively, although the excitatory effect of nicotine also involved mechanisms other than glutamate receptors.

## Discussion

Converging evidence indicates that the LHb is activated by aversive stimuli^36^. Nicotine can have strong aversive effects^37^, which can play an important role in promoting nicotine addiction^1^,^2^. In keeping with this, we found that nicotine strongly activated LHb neurons *in vitro*, as has been observed *in vivo*^38^; *in vivo*, LHb activation of RMTg neurons would in turn inhibit midbrain dopamine (DA) neurons and thus contribute to aversion^12^,^39^. In addition, we found that the actions of nicotine in LHb brain slice were mediated by distinct nAChR subtypes. Pharmacology and α6*-knockout experiments suggested that α6*-nAChRs played a prominent role in the sustained enhancement of LHb firing and glutamate release, while DHβE-sensitive nAChRs, likely reflecting α4β2-nAChRs, were important for the transient increase in GABAergic transmission. Our results show that different nAChR subtypes played a significant role in the regulation of LHb activity, which may contribute to both nicotine aversion and reward.

At different concentrations found in the blood of human smokers (25–444 nM)^31^, nicotine acted at diverse nAChR subtypes to modulate LHb activity in brain slices from rats. Low concentrations (EC_50_ 20–50 nM) potentiated IPSCs via DHβE sensitive (α4β2) nAChRs, leading to rapid but only brief hyperpolarization, consistent with α4β2-nAChR’s propensity to desensitization^40^,^41^,^42^. Higher nicotine concentrations (EC_50_ ~200 nM) elicited a more sustained increase in glutamate release via MII-sensitive (α6* ± α3β2)- and MLA-sensitive (α7)-containing nAChRs; pharmacology and knockout experiments combined suggest a particular role for α6*-nAChRs in nicotine excitation of LHb neurons. Nicotine’s actions on synaptic transmissions depended in part on calcium and TTX-sensitive sodium channels. At higher concentrations (EC_50_ ~400 nM), nicotine directly depolarized LHb neurons via MII- and DHβE-sensitive nAChRs but not by MLA-sensitive nAChRs. Similarly, the nicotine-induced increase in firing was sensitive to ΜΙΙ, MLA and DHβE. The EC_50 _of nicotine for enhancing firing (~600 nM) was about 1.5 fold greater than the EC_50_ of the nicotine-induced current responses. The difference between the EC_50_ for nicotine enhancement of firing and depolarization may reflect the presence of different nAChRs at presynaptic terminals versus postsynaptic neurons in LHb.

Genetic variations in the CHRNA6-CHRNB3 gene cluster increase vulnerability to tobacco smoking^43^,^44^,^45^. α-CTx-MII infusion into α6*-nAChRs-expressing mesolimbic regions (midbrain and nucleus accumbens)^46^,^47^,^48^ decreases nicotine self-administration by rats^21^,^25^. Our findings show that the LHb is another critical brain region for α6*-nAChR-mediated actions for nicotine. First, global disruption of α6-nAChRs increase basal mEPSC frequency as well as firing rate, indicating α6-nAChRs is a critical regulator of LHb neuronal activity. Second, although the MHb also expresses many nAChRs^32^ and projects to LHb^26^, the effects we observed probably originated mainly in the LHb, since α6*-mediated LHb currents were elicited by local applications of ACh or by bath applications of nicotine in the presence of TTX, and persisted when the MHb was cut away from the LHb. Moreover, nicotinic excitation of MHb neurons *in vitro*^49^ does not require α6*-nAChRs^8^. Previous immunoreactivity studies^28^ suggest that α6-nAChRs are mainly expressed in the ventral inferior MHb, with little expression in the LHb. In the current study, using the patch-clamp electrophysiology technique, one of the most sensitive ways to detect functional activity of receptors, we were able to detect that nicotine modulated physiological activity of LHb neurons through action of nAChRs containing the α6 subunit.

Nicotine has both negative and positive motivational effects which contribute to its abuse potential^1^. Here we demonstrate that nicotine, acting through α6*-nAChRs, depolarizes LHb neurons and enhances glutamatergic signaling. Manipulating LHb activity, especially through α6*-nAChRs, may therefore be of great value in treating nicotine addiction. In addition, we have shown that, over a wide range of concentrations (10 nM–100 μM), nicotine robustly activates LHb neurons. In contrast, nicotine at < 100 nM has no significant effects on DA neurons in the ventral tegmental area (VTA); only at ≥250 nM does nicotine increase DA neuronal firing and burst activity^50^,^51^. Interestingly, once sufficient nicotine concentrations are reached, nicotine has a stronger effect on VTA-DA cells than on LHb neurons. For example, we found that nicotine at 1 μM, a concentration within the range of peak blood levels in smokers, increased VTA-DA neuronal discharges by an average of 2.32 ± 0.4 (*n* = 7) fold *in vitro* (see also ref. 52). This is in agreement with other studies^53^, and was significantly greater than the 1.52 ± 0.07 (*n* = 13) fold increase in firing of LHb neurons (*p* = 0.017, unpaired *t*-test). Overall, the finding that nicotine at low concentrations activates LHb but not DA cells indicates that the LHb is exquisitely sensitive to the aversive effects of nicotine.

In addition to the LHb directly encoding aversive stimuli, activation of LHb neurons may contribute to aversion by inhibiting DA neurons indirectly via the RMTg^54^,^55^. Like the LHb, the RMTg is activated by aversive stimuli. Also, our findings that nicotine excites LHb glutamatergic neurons agree with recent studies showing that nicotine increases presynaptic glutamate release onto RMTg neurons^12^,^39^. Furthermore, a previous electrophysiological study in anesthetized rats showed that systemic administration of nicotine led to VTA-DA neuron activation which was preceded by a short-lasting inhibition^56^. We speculate that this inhibition of DA neurons could be caused by nicotine-induced excitation of LHb neurons, which activates the RMTg to inhibit DA cells. Emerging data also demonstrates that activation of the LHb-to-RMTg pathway induces acute avoidance^9^, and that activation of the Hb-IPN pathway reduces nicotine intake^57^. The Hb and its targets may also be important for symptoms of nicotine withdrawal, since inhibition of nAChRs in the Hb attenuates withdrawal symptoms^6^. In agreement, chronic nicotine exposure causes a hypodopaminergic state^58^, which may mediate the effects of nicotine withdrawal. Thus, reducing Hb-mediated inhibition of VTA DA neurons could be a useful strategy to relieve these withdrawal symptoms. These findings indicate that the LHb likely plays a critical role in different aspects of nicotine addiction.

Taken together, our data suggest that different subtypes of functional nAChRs are situated both presynaptically on local afferents innervating LHb neurons and postsynaptically on the somatodendritic regions of these neurons. By activating these receptors, nicotine likely enhance the output of LHb neurons and, in this way, contribute to the aversive properties of nicotine. Thus, direct nicotine action within the LHb via the pathways we have identified may represent a mechanism through which nicotine-related aversion could contribute to nicotine addiction.

## Methods

### Animals

The Institutional Animal Care and Use Committee of Rutgers, Rutgers, the State University of New Jersey, and the Barrow Neurological Institute, St. Joseph’s Hospital and Medical Center, approved all procedures. All methods were performed in accordance with the relevant guidelines and regulations. Animals were housed under standard conditions at 22–24 °C, 50–60% humidity, and a 12 h light/dark cycle. For electrophysiological experiments, we used Sprague-Dawley rats (at postnatal days 20–40) and C57BL/6 (B6) mice [wild-type (WT) or nAChR α6 subunit knock-out (α6 KO) mice, at postnatal days 16–23]. As data obtained from either sex did not differ significantly, they were pooled.

### Brain slice preparation and electrophysiology

Brain slices were prepared as described earlier^59^. Animals were anesthetised with ketamine/xylazine (80 mg/5 mg/kg i.p.) and then euthanized by decapitation. Coronal epithalamic slices (250 μm) were cut in ice-cold glycerol-based artificial cerebrospinal fluid (GACSF) containing (in mM): 252 glycerol, 2.5 KCl, 1.25 NaH_2_PO_4_, 1 MgCl_2_, 2 CaCl_2_, 25 NaHCO_3_, 0.3 L-ascorbate, and 11 glucose, and saturated with 95%O_2_/5%CO_2_ (carbogen). Slices were then incubated >1-hr at 24–25 °C in carbogenated ACSF of similar composition as GACSF, but with 126 mM NaCl replacing glycerol. In a low Ca^2+^ solution, CaCl_2_ was replaced by an equivalent amount of MgCl_2_. The experiments were performed on slices in submerged in a chamber perfused with ACSF at ~33 °C, at 1.5–2 ml/min. Electrical signals were recorded using an Axon 200B or 700A amplifier and Clampfit 10.3 (Molecular Devices, Union City, CA), filtered at 2 kHz and sampled at 5 kHz. Patch pipettes (6–8 MΩ) for voltage-clamp recordings contained (in mM): 140 Cs-methanesulfonate, 5 KCl, 2 MgCl_2_, 10 HEPES, 2 MgATP, 0.2 NaGTP, pH 7.2. The internal solution for current-clamp recordings was similar except that 140 mM K-gluconate replaced Cs-methanesulfonate. IPSCs were recorded at +40 mV in the presence of AP5 (50 μM), DNQX (20 μM) and strychnine (1 μM) to block NMDA, AMPA and glycine receptors. EPSCs were recorded at −70 mV with strychnine (1 μM) and gabazine (10 μM) to block glycine and GABA_A_ receptors. Miniature EPSCs were recorded in the presence of 0.5 μM tetrodotoxin (TTX). PSCs were evoked with a bipolar nichrome electrode within 200 μm from the recorded neurons, applying 100–200 μs stimuli at 0.05 Hz. After obtaining an input/output curve, stimulation was set to a level eliciting 20–30% of maximal currents. Spontaneous firing was recorded by the loose-patch cell-attached technique, allowing long-lasting recordings without perturbing the cytoplasmic contents, and in whole-cell mode to measure membrane potential and input resistance. Currents-induced by nicotine or acetylcholine (ACh) were recorded in TTX, AP5, DNQX, gabazine, and strychnine. Compounds were applied by bath perfusion, except acetylcholine (30 μM, with 0.5 μM atropine to block muscarinic receptors) which was ejected by pressurized air from a micropipette placed near the recorded cell^33^.

### Drugs

We purchased common salts and [L-1-methyl-2-(3-pyridyl) pyrrolidine] freebase (nicotine), 6,7-dinitroquinoxaline-2,3-dione (DNQX), DL-2-amino-5-phosphono-valeric acid (AP5), strychnine, mecamylamine hydrochloride, methyllycaconitine, gabazine, atropine, acetylcholine, CdCl_2_, TTX from Sigma (St Louis, MO), and dihydro-β-erythroidine hydrobromide from Santa Cruz Biotechnology (Santa Cruz, CA). α-conotoxin MII (α-CTx-MII) and MII [H9A; L15A] were synthesized^30^.

### Data Analysis and Statistics

Baseline electrophysiological data were recorded for 5–10 min, before drug superfusion and during the washout. To calculate the percent change in EPSCs/IPSCs/firing frequency or amplitude for a given cell, recordings during the initial control period were averaged and normalized to 100%. eIPSC and eEPSC amplitudes were calculated by averaging the peak current from six sweeps during baseline and during each drug application. For the measurement of inward currents induced by bath application of nicotine, traces were filtered at 10 Hz, and the means of 30-s baseline before nicotine application and 30 s during the maximal effects of nicotine were calculated and subtracted to give the magnitude of the nicotine current. The peak of nicotinic currents induced by puffing ACh onto neuronal somata was measured after the traces were filtered at 500 Hz. All values given in the text and figures indicate mean ± S.E.M. Statistical significance was assessed by a two-tailed Student’s *t* test, a one-way ANOVA with a Tukey’s *post hoc* test for multiple group comparisons, or a Kolmogorov-Smirnov (K-S) test. Dose-response data were fitted to the logistic equation: y = 100x^α^/(x^α^ + x_o_^α^), where y is the percentage change, x is the concentration of nicotine, α the slope parameter, and x_o_ the nicotine concentration which induces a half-maximal change.

## Additional Information

**How to cite this article**: Zuo, W. *et al*. Nicotine regulates activity of lateral habenula neurons via presynaptic and postsynaptic mechanisms. *Sci. Rep.* **6**, 32937; doi: 10.1038/srep32937 (2016).

## Figures and Tables

**Table 1:** GABA or/and glutamate transmissions mediate nicotine–induced changes in firing of LHb neurons.

Nicotine-induced change of firing rate	ACSF	+Gabazine	+DNQX + AP5	+Gabazine + DNQX +AP5
Inhibition (% of baseline)	90.4 ± 3.8 (*n* = 8)	101.2 ± 1.0* (*n* = 8)	86.9 ± 3.5 (*n* = 8)	103.4 ± 2.5* (*n* = 8)
Excitation (% of baseline)	149.8 ± 7.3 (*n* = 14)	155.4 ± 6.8 (*n* = 8)	122.4 ± 3.5* (*n* = 8)	126.5 ± 3.9* (*n* = 8)

Nicotine (100 nM)-induced inhibition of spontaneous firing was abolished by the GABA_A_ receptor antagonist gabazine, while nicotine-induced acceleration of firing was partly attenuated by glutamate receptor antagonists DNQX+AP5. *p < 0.05, One-way ANOVA followed by Tukey’s multiple comparison test for nicotine application in ACSF vs nicotine plus these blockers.
